# Validation of a semiautomated volumetric approach for fetal neurosonography using 5DCNS+ in clinical data from > 1100 consecutive pregnancies

**DOI:** 10.1007/s00381-020-04607-5

**Published:** 2020-04-30

**Authors:** Amrei Welp, Michael Gembicki, Achim Rody, Jan Weichert

**Affiliations:** Department of Obstetrics and Gynecology, Division of Prenatal Medicine, University Hospital of Schleswig-Holstein, Campus Luebeck, Ratzeburger Allee 160, 23538 Luebeck, Germany

**Keywords:** Brain, 3D ultrasound, Semiautomatic reconstruction, Cortical development

## Abstract

**Objective:**

The aim of this study was to evaluate the validity of a semiautomated volumetric approach (5DCNS+) for the detailed assessment of the fetal brain in a clinical setting.

**Methods:**

Stored 3D volumes of > 1100 consecutive 2nd and 3rd trimester pregnancies (range 15–36 gestational weeks) were analyzed using a workflow-based volumetric approach 5DCNS+, enabling semiautomated reconstruction of diagnostic planes of the fetal central nervous system (CNS). All 3D data sets were examined for plane accuracy, the need for manual adjustment, and fetal-maternal characteristics affecting successful plane reconstruction. We also examined the potential of these standardized views to give additional information on proper gyration and sulci formation with advancing gestation.

**Results:**

Based on our data, we were able to show that gestational age with an OR of 1.085 (95% CI 1.041–1.132) and maternal BMI with an OR of 1.022 (95% CI 1.041–1.054) only had a slight impact on the number of manual adjustments needed to reconstruct the complete volume, while maternal age and fetal position during acquisition (*p* = 0.260) did not have a significant effect. For the vast majority (958/1019; 94%) of volumes, using 5DCNS+ resulted in proper reconstruction of all nine diagnostic planes. In less than 1% (89/9171 planes) of volumes, the program failed to give sufficient information. 5DCNS+ was able to show the onset and changing appearance of CNS folding in a detailed and timely manner (lateral/parietooccipital sulcus formation seen in < 65% at 16–17 gestational weeks vs. 94.6% at 19 weeks).

**Conclusions:**

The 5DCNS+ method provides a reliable algorithm to produce detailed, 3D volume–based assessments of fetal CNS integrity through a standardized reconstruction of the orthogonal diagnostic planes. The method further gives valid and reproducible information regarding ongoing cortical development retrieved from these volume sets that might aid in earlier in utero recognition of subtle structural CNS anomalies.

**Electronic supplementary material:**

The online version of this article (10.1007/s00381-020-04607-5) contains supplementary material, which is available to authorized users.

## Introduction

The diagnosis of fetal cerebral anomalies can be devastating for parents-to-be because most severe central nervous system (CNS) malformations lead to in utero demise or severe neuromotoric sequelae. Although ultrasound techniques have markedly improved over past decades, the prenatal diagnosis of fetal cerebral anomalies remains challenging. The detection rates of gross fetal CNS anomalies via ultrasound are 96.6% (anencephaly), 82.5% (spina bifida), and 73.4% (hydrocephaly) [8]. Similar results have been reported elsewhere [3, 17]. For more rare and subtle lesions in CNS anatomic integrity, including disorders of cortical development like lissencephaly or schizencephaly, antenatal diagnosis is not straightforward and detection rates are rather low, thereby underscoring the urgent need of a skilled sonographic assessment both in high- and low-risk cohorts.

The benefits of a three-dimensional (3D) comprehensive examination of the brain have been clearly demonstrated. However, difficult fetal orientation within the 3D volume, non-intuitive manipulation along the x, y, z planes, and, most strikingly, the lack of standardization perceived to be challenging. The ability to obtain correct diagnostic planes for a detailed neurosonogram from a 3D volume is operator-dependent and directly related to the expertise of the examiner 13]. Recent studies were able to show that a semiautomated approach might overcome these shortcomings [25]. Rizzo et al. reported the potential for the 5DCNS+ software to be an accurate and reliable technique for fetal neurosonography [22–25].

The primary objective of this study was to evaluate the clinical use and validity of 5DCNS+ as a postprocessing tool, enabling the correct display of all diagnostic planes of the fetal CNS. We further investigated the applicability of 5DCNS+ to assess details of cortical development throughout advancing pregnancy with a particular focus on gyration and operculization.

## Methods

This study included 1110 volume data sets obtained by two operators with expertise in prenatal ultrasound from singleton pregnancies acquired between April 2015 and April 2019 at a single tertiary referral center. Fetuses with abnormal CNS anatomy were excluded from final analysis. All examinations were performed with WS80A Elite ultrasound equipment (Samsung HME, Korea) equipped with a 1–8 MHz curved transducer (S-Vue™- Transducer CV1-8A) using the trans-abdominal technique. The referral base was comprised of a mixed-risk cohort (women at either high or low risk for fetal abnormalities).

**Fig. 1:**
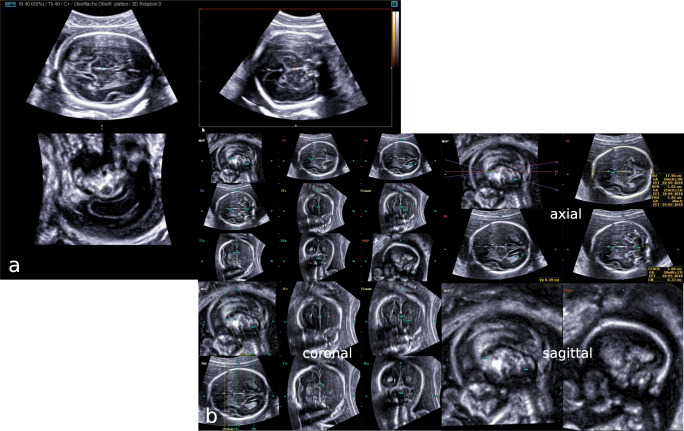
Resulting multiplanar view of the fetal CNS at 23 gestational weeks after successful volume acquisition (step 1) (**a**). Following orthogonal plane reorientation along the x-, y-, z-axis to align the falx cerebri horizontally, the 5DCNS+ tool has been applied by simply drawing a straight line between the thalami and the cavum septi pellucidi (step 2). The resulting diagnostic planes may be displayed all together or grouped in axial, coronal, and sagittal planes as intended by the operator (**b**)

Prior to volume acquisition, all fetuses underwent a conventional 2D ultrasound examination of the CNS as part of the anatomic survey. Subsequently, image adjustment using high-resolution magnification was necessary to have the fetal head occupy approximately 75% of the screen in an axial (transverse) view. Originating from this axial view, which corresponds to the transthalamic plane that is routinely used for basic CNS assessment and biometry, all 3D volumes were acquired in the absence of fetal movements (step 1). The volume acquisition angle of the US sweep was set between 45° and 65° according to the gestational age to include the entire fetal brain in the volume. In the resulting triplanar view, the orthogonal planes were reorientated in order to align the falx cerebri horizontally (A and B plane; video clip 1 and 2). Then, to run the 5DCNS+ tool properly during step 2, two reference points as local constraints that define the correct cutting plane were marked manually—the 1st seed in the middle of the thalami and the 2nd seed within the cavum septi pellucidi. Finally, the workflow implemented in the 5DCNS+ algorithm allows for semiautomatic reconstruction of all nine diagnostic CNS planes (comprising a complete fetal neurosonogram). Then, the resulting three axial, four coronal, and two sagittal diagnostic planes can be displayed, reviewed, and manipulated separately or in groups (Fig. 1; see also supplemental files).

**Fig. 2:**
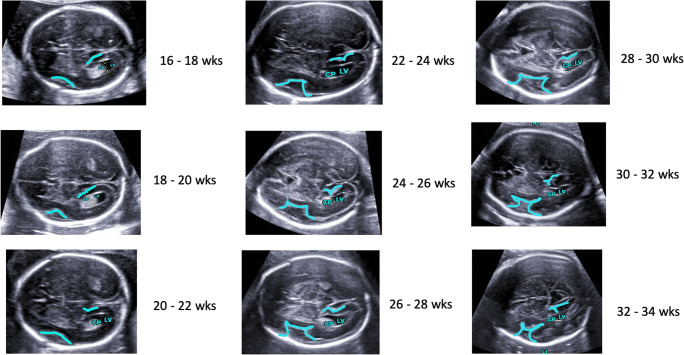
Illustration of additional information that can be taken from the reconstructed diagnostic planes. Progress of CNS folding with advancing pregnancy (16 to 34 weeks) demonstrated in axial views retrieved from volume reconstruction using the semiautomized 5DCNS+ technique

All volumes obtained by 5DCNS+ technique were analyzed and judged in terms of adequately displayed planes, the need for manual adjustment, and accuracy of program-derived biometric measurements (and compared with manually calculated measures). For detailed assessment of the fetal cortex and insula region, the axial transventricular plane was abstracted and augmented before evaluation. The changing shape of both the sylvian fissure and parietooccipital sulcus was highlighted as illustrated in Fig. 2.

Only those 3D volumes from examinations between the 15th and 36th week of pregnancy were included to ensure an accurate evaluation (at earlier and later gestational ages, a detailed evaluation can be compromised due to the not-yet-detectable landmarks of the developing brain or by shadowing artifacts caused by progress in bony calcification, respectively).

All statistical tests were run through Sigmaplot (Version 12.0, SyStat, USA). To evaluate a possible impact of fetal and maternal factors, multiple logistic regression was used (*p* values < 0.05 were considered as statistically significant).

## Results

**Fig. 3:**
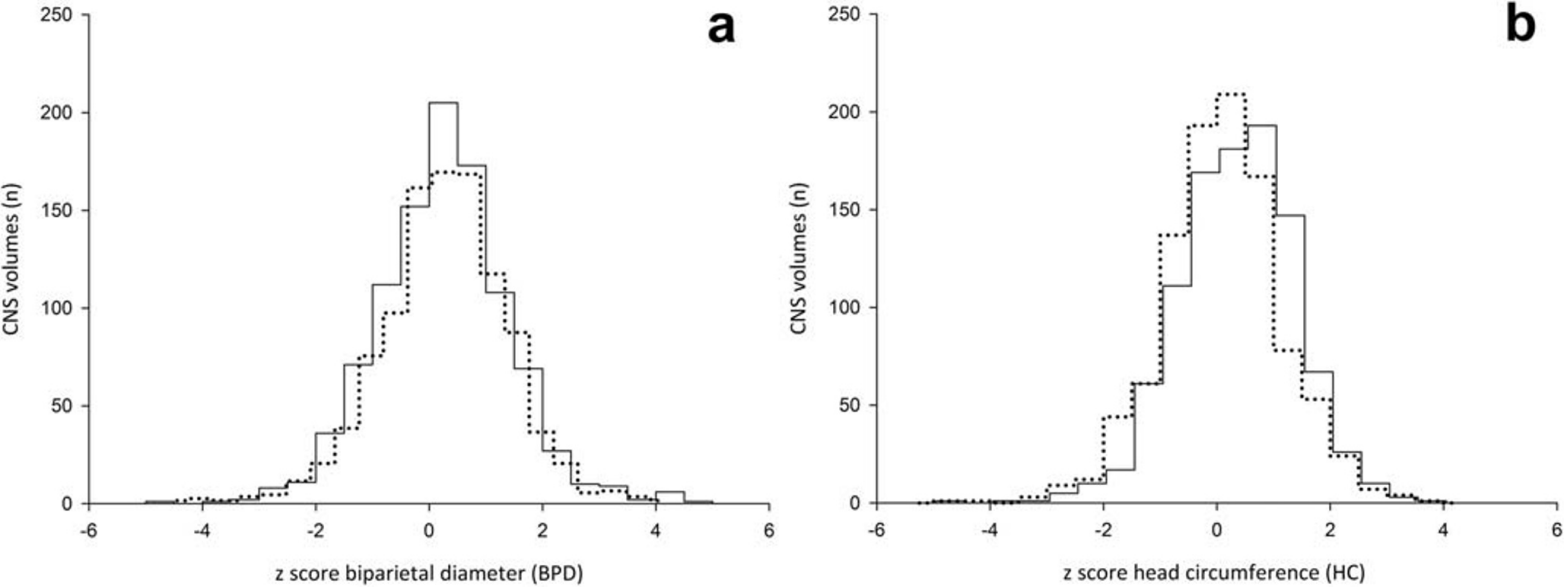
Histogram of the differences of biometric measurement of head circumference (HC; **a**) and biparietal diameter (BPD; **b**) acquired either semiautomatically by the 5DCNS+ tool (dotted line) or manually calculated (straight line)

A total of 1019 cases were enrolled in the final analysis after excluding abnormal cases and volumes acquired during first trimester. The mean gestational age was 23.2 weeks (ranging from 15.0 to 36.4 weeks), the mean maternal age was 32.2 years (ranging from 16 to 53 years), and the mean body mass index was 25.9 (ranging from 16.7 to 49 kg/m^2^). One to four separate volumes were obtained per patient (a mean of 1.1 exams) (Table 1). Pregnancies with proven CNS anomalies were excluded. Regarding the accuracy of semiautomatized biometric measurement of biparietal diameter and head circumference compared with manually calculated dimensions (expressed as z-scores) we noticed a slight difference between both methods as shown in Fig. 3. Interestingly, subgroup analysis revealed that this difference reached statistical significance only beyond 25 completed weeks.

**Table 1:** General characteristics of the study population

Variable	Mean [range]
Maternal age (years)	32.2 [16–53]
Maternal body mass index (kg/m^2^)	25.9 [16.7–49]
Gestational age at ultrasound examination (weeks)	23.2 [15.0–36.4]

To determine fetal and maternal factors that might complicate the feasibility of the 5DCNS+ tool due to a potential confounding impact, maternal age, gestational age, maternal body mass index, and fetal position in utero were analyzed with regard to a need to manually adjust the plane. Multiple logistic regression revealed a small impact of gestational age and body mass index on additional volume manipulation for successful plane reconstruction. Maternal age seemed to have no significant effect on proper volume postprocessing (Table 2).

**Table 2:** Impact of maternal and fetal factors

Variable	Odds ratio	95% confidence interval
Maternal age	0.981	0.953–1.009
Maternal body mass index	1.022	1.041–1.054
Gestational age at ultrasound examination	1.085	1.041–1.132

In a second step, the effect of fetal position at time of volume acquisition was evaluated. There were 676 fetuses in a cephalic position, 71 fetuses in a transverse position, and 273 fetuses in a breech position, respectively. The Kruskal one-way analysis of variance (ANOVA) on rank showed no significant differences among all three groups (*p* = 0.260).

In 999/1019 (98%) of the volumes, ≥ 8 diagnostic planes were sufficiently visualized, and in 958/1019 (94%) of volumes, all nine planes were sufficiently visualized. Manual adjustment was needed in at least one diagnostic plane in nearly half of the cases, and more than two diagnostic planes had to be adjusted in 206/1019 cases (20.4%). Examining the volumes in detail showed differences between the orthogonal planes. In general, adjustment to the coronal planes was required less often compared with adjustment to the axial planes (492 vs. 628). A sufficient reconstruction of the midsagittal plane in all 1019 enrolled cases was noticed, whereas the axial transventricular plane was not successfully reconstructed in 244 cases that needed an additional manual adjustment. The dropout rate (number of planes with an unsatisfactory location after manual adjustment) was small. Of 9171 planes in total, 89 planes (0.97%) could not be obtained properly. While the parasagittal plane had 55 dropouts, the remaining eight diagnostic planes had less than ten dropouts (Table 3).

**Table 3:** Frequency of satisfactory views (%) according to type of CNS plane

	No dropout	No dropout or adjustment
Axial planes		
Transventricular plane	99.6	78.5
Transthalamic plane	99.9	83.3
Transcerebellar plane	99.9	75.9
Coronal planes		
Transfrontal plane	99.2	77.2
Transcaudate plane	99.7	90.7
Transthalamic plane	99.8	98.3
Transcerebellar plane	98.9	83.1
Sagittal planes		
Midsagittal plane	99.6	99.6
Parasagittal plane	94.6	77.5

In addition to the complete and satisfactory reconstruction of the neurosonographic planes, morphologic assessment revealed that in 860 of 1019 volumes, the typical appearance of anatomic structures could be clearly identified. Particularly in an axial (transventricular) view, a profound and detailed examination of the sylvian fissure, the parietooccipital sulcus and the calcarine sulcus could readily be performed. A thorough judgment regarding the completion of developmental milestones in a timely manner was feasible. The same method was applied to the process of operculization (the differentiation of the lateral sulcus with programmed invagination of the insula), which was sufficiently demonstrated using 5DCNS+ (Fig. 2). The latter was visualized in two-thirds of cases assessed at 16–17 weeks (64.3%), which rapidly changed to 94.6% at 19 weeks and 100% at 20 weeks.

## Discussion

Although a large number of congenital anomalies of the fetal CNS can be consistently recognized during an anatomic ultrasound survey, there is still a marked variation in detection rates. This difference might be mainly attributed to the varying prevalence of CNS anomalies, the (developmental) stage-dependent visualization, and the expertise of the examiner.

According to recent data published by the EUROCAT working group, the reported prevalence of cerebral anomalies increased within a decade, potentially because of improved antenatal detection rates [16]. On the other hand, the accuracy of a prenatal diagnosis remains heterogeneous, hampering individually tailored parental counseling [26]. Prerequisites for detailed fetal neurosonographic work-ups include an exact understanding of the anatomic integrity of the normal brain, the correct delineation of all diagnostic planes, and the ability to instantaneously retrieve valuable information on CNS abnormalities during conventional 2D ultrasound examinations. These operator-dependent tasks may hamper the precise and early recognition of more subtle changes in cerebral anatomy, which impacts timely and informed decision making on extended genetic testing, amenability to fetal surgery, or termination of the pregnancy. The diagnostic value of volumetric 3D approaches for advanced assessments of the fetal CNS has been demonstrated in the literature [7, 29]. With the recent introduction of the intelligent semiautomatic 5DCNS/5DCNS+ technology, a 3D volume assessment standardization via workflow-based axial, coronal, and sagittal plane reconstruction has been achieved, contributing to a further reduction of intra- and interobserver variability.

Based on our results, we were able to show that using 5DCNS+ after a 3D volume acquisition allows for rapid and reliable plane reconstruction in 98% of all pregnancies, which is in line with previously published data. In 958/1019 cases, a detailed neurosonogram [4, 12, 28] including all nine diagnostic planes as recommended by international guidelines [11] was obtained. Initial reports have emphasized the feasibility and potential for modulating the efficiency of qualitative and quantitative CNS assessments by this method [22, 25]. We further showed that using 5DCNS+ results in neither additional time for completion of the anatomic survey nor any change in established workflow. The average time required for the semiautomatic reconstruction was less than 40 to 50 s and could be readily integrated in the examination protocol. As image segmentation, which is the methodological basis of quite a number of automated volumetric approaches, allows for axial plane extraction from the original volume needed for an automated biometry of the CNS, it may further aid in completion of the anatomic survey in a time-saving manner. However, the results derived from algorithm-based calculation in advanced pregnancy should be interpreted with caution as these measures tend to be slightly underestimated as shown in Fig. 3. Examining the relevance of fetal-maternal factors such as maternal age and body mass index, as well as gestational age and fetal position in utero at acquisition, revealed in insignificant or a weak impact on final image reconstruction via 5DCNS+. This result might seem in contrast to previous studies; Rizzo et al. stated that 5.5% of CNS volumes assessed were of limited clinical value because of poor image quality [23, 24]. Maiz et al. reported a direct link of diminished image quality and both gestational age and fetal head position [13]. On the other hand, the outcome might illustrate the improvements of volume postprocessing using the 5DCNS+ technique.

Imaging technologies for antenatal cerebral anatomy assessments have substantially evolved over the last two decades, allowing more detailed descriptions of the ongoing process of gyration and sulci formation. To ascertain normal vs. abnormal cortical development (due to cerebral malformations or as sequelae of intrauterine growth restriction [10] or extracerebral diseases (e.g., congenital heart disease) [18]), there have been several attempts in the literature to systematically evaluate the physiological changes in the appearance of primary fissures. It has recently been shown that the development of the sylvian fissure, a landmark of gyration, can be identified on a 2D ultrasound from 16 gestational weeks onward [21, 27]. Accordingly, an accurate grading of the time-dependent cortical folding for other primary fissures such as the parietooccipital fissure, calcarine fissure, and the cingulate sulcus is feasible with obligate visualization from 23 and 24 weeks onward [1, 5]. There are also technical issues in the comprehensive assessment of the sylvian fissure in the same cutting plane and a lack of standardization in both plane reconstruction and the quantification of spatial-cerebral relationships [19]. In 2015, two publications reported the reliability of multiplanar 3D ultrasounds for systematic (longitudinal) assessments of cortical differentiation, confirming prior studies [2, 6, 9, 14, 15]. Gindes et al. demonstrated that the sylvian fissure grows asymmetrically in an anterior-posterior direction rather than laterally or in the inferior-superior direction. Very recently, reference charts of the sylvian fissure angle obtained in strictly coronal views have been published [20], giving early hints towards delayed development seen in most cortical malformations [19]. Our data corroborate and further specify the time pattern of CNS folding because we had 100% visualization of the lateral sulcus and the ongoing process of operculization at 20 completed weeks.

However, there is not widespread, daily use because most examiners lack familiarity with the focused use of 3D ultrasound and because volume navigation is complex. The aforementioned 5DCNS+ is able to simplify thorough 3D volume–based examinations of the fetal brain. Our data suggest that this technology even allows concomitant and highly replicable reconstruction of those diagnostic planes needed for the proper evaluation of cortical differentiation.

The strength of this study is its large sample size of more than 1000 patients scheduled for a targeted anatomic survey. Our study includes in-depth analysis of the clinical applicability of a semiautomated volumetric diagnostic technique, providing detailed insights into how this tool might aid prenatal assessments of complex CNS anatomy.

One of the limitations of the current study is that all volumes were acquired by operators with expertise in fetal neurosonography. Moreover, as a referral center, our patient population is biased. Consequently, the success rate of volume reconstruction might be lower in peripheral centers with less experienced examiners, underscoring the need for training so that volume acquisition itself is no longer the major limiting factor.

In conclusion, 5DCNS+ is an efficient and valuable method to readily provide detailed additional information on the integrity of the fetal CNS. Its clinical applicability has been clearly demonstrated; this workflow-based volumetric approach supports the evaluation of the brain in a standardized manner (1), and it might aid early and systematic assessments of fetal cortical development (2) and potentially allows for the detection of subtle anatomic changes of the CNS (3). Our data illustrate the potential of this technique in helping extract essential information from CNS anatomy and providing a link to postnatal imaging and (re) assessment of the spatial arrangement and severity of fetal anomalies.

However, the reliability and effective sensitivity of 5DCNS+ in the case of minor and major CNS abnormalities have to be addressed in further studies.

## Electronic supplementary material

ESM 1 (MP4 21743 kb)

ESM 2 (MP4 3001 kb)
